# Stone Wool Substrate Cover Incision Impacts on the Root-Zone Water Content, Temperature, and Yield of Tomato Cultures

**DOI:** 10.3389/fpls.2022.875730

**Published:** 2022-06-09

**Authors:** Tae In Ahn, Jung-Seok Yang, Yong-Hoon Im, Young Jik Youn, Ju Young Lee

**Affiliations:** ^1^Smart Farm Research Center, KIST Gangneung Institute of Natural Products, Gangneung, South Korea; ^2^Division of Mechanical System Engineering, Sookmyung Women's University, Seoul, South Korea; ^3^Energy Efficiency Research Division, Korea Institute of Energy Research, Daejeon, South Korea

**Keywords:** stone wool, drainage, water management, hydraulic property, tomato, thermal sensitivity

## Abstract

Standardized cultivation systems are crucial for establishing reproducible agronomic techniques. Especially stone wool-based cultivation is governed by standardized specifications and provides a controllable root-zone environment. However, the effects of stone wool cover incision on root-zone variability have rarely been studied. Therefore, in this study, we focused on the effect of the stone wool cover incision method on environmental variations and their subsequent effects on tomato plant productivity. Stone wool slab plastic covers represent a core component of this substrate system that can potentially affect the performance of water control techniques. We designed a cover incision method to create four different levels of drainage performances that were tested by cultivating tomato plants (*Solanum lycopersicum* “Dafnis”). The water content, root-zone temperature, and dissolved oxygen were measured and analyzed relative to the tomato yield. We found that the incision level with the lowest drainage performance showed a lower air-root zone temperature correlation slope than those of slabs with favorable drainage conditions. Furthermore, these slabs had low dissolved oxygen levels (3.2 mg/L); nevertheless, the tomatoes grown in the slabs with incision level showing the lowest drainage performance had greater fruit yield (6,748 g/plant) than those in the slabs with favorable drainage conditions (6,160 g/plant). Furthermore, the normalized yield separation timing between treatments coincided with the hotter air temperature (27°C average) periods. We noted that manipulating the cover incision process consequently entailed variations in the correlation slope between the air temperature and root-zone temperature in the substrate. Our results reveal another trade-off relationship in the conventional perspective on the drainage performance effects and provide insights into further optimization of crop production and water use in the stone wool-based system.

## 1. Introduction

In contrast to soil-based cultivation, soilless cultures are prepared using solid substrates as rooting media. Especially the stone wool substrate is widely used in horticultural soilless crop production in controlled environment agriculture (Gruda, 2019). Further, it has confined root zone boundaries and rapidly responds to root zone environment manipulations (Sonneveld, 1991; Saha et al., 2008). Hence, the stone wool culture represents a basic production model, and technical frameworks have long been built on stone wool-based systems.

Typically, the monitored variables in a stone wool-based system include transpiration-dependent irrigation, drainage ratio, root-zone water content, and electrical conductivity (EC) (Shin and Son, 2016). In this system, temporal water control occurs in three major phases (Lee, 2010; Ahn et al., 2020): controlled irrigation commences post sunrise (to compensate for crop water use during the day and the previous night); water is discharged after substrate field capacity is reached; and the controlled irrigation ends around sunset. Furthermore, commercially available sensors such as for measuring radiation, infrared, gutter weight, and substrate moisture, can be utilized for on-farm applications (Shin and Son, 2016; de Koning and Tsafaras, 2017; Montesano et al., 2018). In all, stone wool-based agronomic systems have greatly improved the productivity of soilless crop farming.

Precision control techniques can be applied for the stone wool-based system as it was built on the standardized specifications of the root-zone environment. However, to maximize its performance, it is critical to understand the effect of changing certain parameters of the stone wool substrate. The stone wool substrate usually accommodates three to six plants per slab depending on the cultivation conditions (Jensen, 1997; Shin and Son, 2016), and nutrient and water management processes are carried out in a volume of approximately only 0.01 m^3^ with a plastic cover wrapping (Sonneveld, 1991; Bussell and Mckennie, 2004). A reduction in the size of the root zone volume lowers the buffering capacity of root environment and requires more stringent management of the system parameters (Van Noordwijk, 1990). Under such conditions, fine-tuning system parameters such as physical shape of the stone wool slab and dripper positions may significantly impact the root-zone environment and yield responses (De Rijck and Schrevens, 1998; Bar-yosef, 2008; Saiful Islam et al., 2008). Indeed, previous studies have analyzed the effects of stone-wool system parameters on the hydraulic properties of the stone wool substrate (Bougoul et al., 2005; Bougoul and Boulard, 2006). Stone wool cover is also of critical importance for drainage and gas exchange performance; however, the cover incision effects on the root-zone environment have rarely been studied as an optimizing factor under stone wool-based water management regime.

In our study, we used the stone wool substrate to test the effect of the plastic cover incision on the root-zone environment and crop yield. Stone wool substrates are formed into slabs and wrapped with a plastic cover. The slabs are used with the cover, and the way the drain hole is cut into the plastic cover can affect the liquid and gas exchange between the root and outside environment. Thus, users generally follow the standard procedures recommended by the manufacturer's guidelines, which primarily focus on favorable drainage performance (Grodan, 2022). Further, root zone temperature is another crucial environmental factor affecting plant growth (Zhao et al., 2021). The stone wool system has lower complexity and fewer variables than the soil system. However, the breathability of the cover, oxygen diffusion in the root zone, drainage rate, thermal condition, and water retention properties of the stone wool system could be sensitively intertwined due to its low buffering capacity, and minor modifications of these components could entail significant environmental variations. The potential cascading effects of modifying these variables can be significant to the root zone environment variability. Thus, in this study, we investigated the series of environmental changes caused by the variations in the slab cover incision levels and their subsequent effect on crop productivity. To our knowledge, this is the first study to investigate the association between slab cover incision and water, thermal, and dissolved oxygen parameters, and the subsequent effect of their variations on crop production. In a greenhouse experiment, four different levels of incisions were made in the stone wool slabs resulting in different levels of cover openness for tomato cultivation. Thereafter, we monitored the root-zone temperature, water content, and dissolved oxygen in the stone wool substrate water, along with the tomato yield.

## 2. Materials and Methods

### 2.1. Greenhouse Experiment

**Figure 1:**
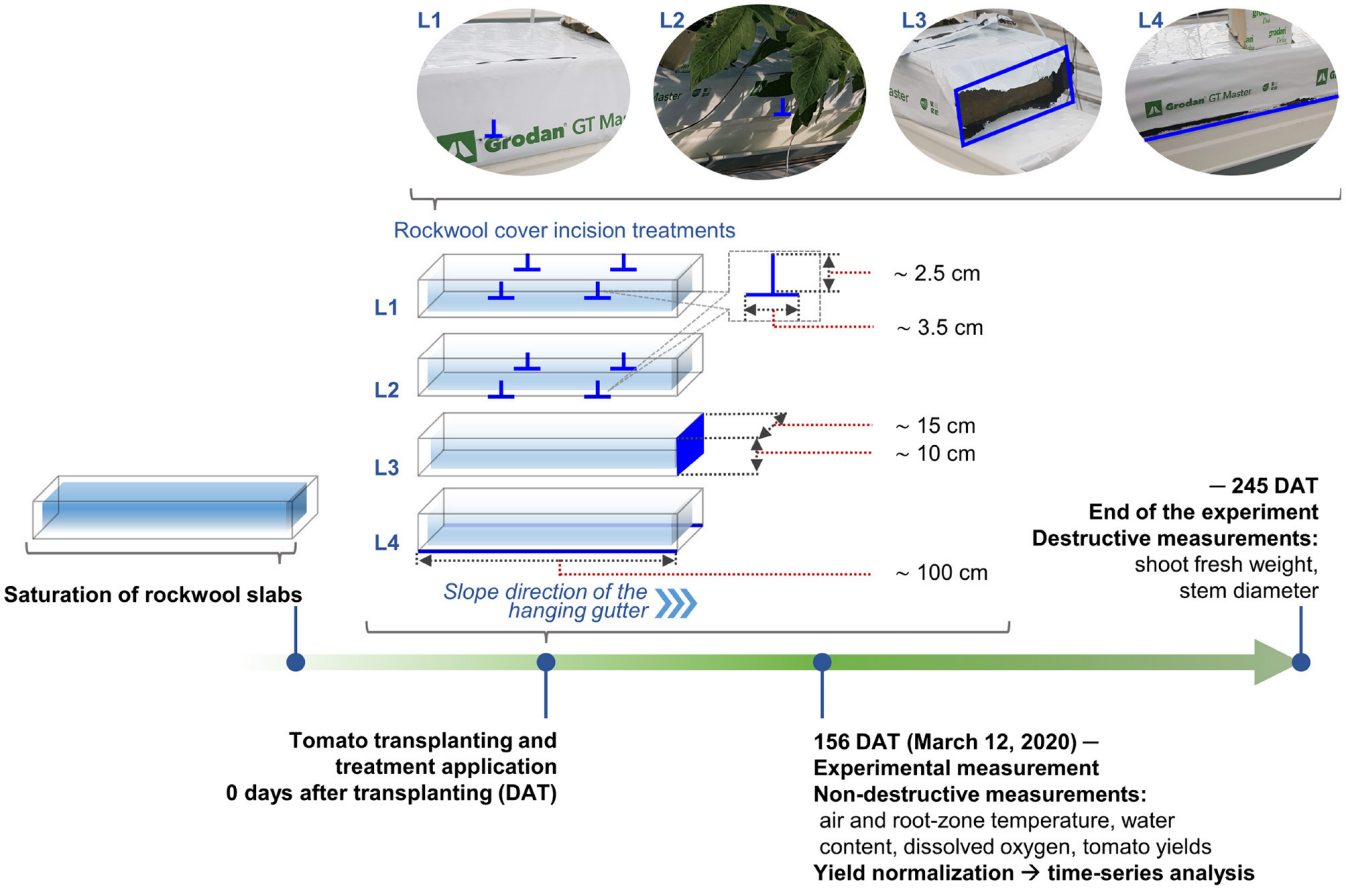
Workflow of the stone wool cover incision treatment and analysis.

Cultivation experiments were conducted in an experimental plastic greenhouse at the KIST Gangneung Institute of Natural Products (37.8°N, 128.8°E). Six tomato plants (*Solanum lycopersicum* “Dafnis”) were cultivated in stone wool slabs (100 × 15 × 10 cm; Grodan GT Master, Grodan, The Netherlands) placed in a hanging gutter of ~9.6 m with a planting density of 2.67 plants m^−2^. Irrigation was controlled with an automatic drip irrigation system using an integrated solar radiation method. Daily irrigation was regularly adjusted so that the daily drainage ratio of the general cultivation lines, present in the same greenhouse, that were not used for experimental treatment, was maintained at about 30% of the daily irrigation amount. Thus, all experimental gutter lines were irrigated with same amount of water. Tomato crops were planted in stone wool slabs with cover incisions on October 8, 2019 (Figure 1). The electrical conductivity and pH of the irrigated nutrient solution was controlled at 2.7 mS m^−1^, and 5.5–6.5, respectively.

### 2.2. Stone Wool Slab Cover Incision Treatments

Four levels of stone wool slab cover incisions were designed (Figure 1) as follows: (1) four-point incision at one-third of the height of the slab (L1), (2) four-point incision at the bottom of the slab (L2), (3) full single-sided incision in the horizontal direction of the slab (L3), and (4) full double-sided incision in the longitudinal direction of the slab (L4). For the incision level and positions of L2 and L4 (Grodan, 2022), we referred to the manual provided by the stone wool slab manufacturer; the remaining incision treatments (L1 and L3) were designed accordingly to indicate the highest and lowest drainage performance, respectively. The numeric details about the incision levels are presented in Figure 1. The slab cover drainage performance and breathability of each treatment were manipulated in the following order: L1 < L2 < L3 < L4. All stone wool slabs were saturated with the standard nutrient solution before tomato transplantation and the cover incisions were made right after transplanting.

### 2.3. Measurements

Experimental measurements were performed from March 12, 2020, 156 days after transplanting (DAT) to 245 DAT (Figure 1). Biomass and yield measurements were performed to compare plant growth between the different stone wool cover incision treatments. Total yield per plant, fruit weight, and the number of fruits per plant were measured after harvest in 20 plants of each treatment. Fruits displaying nearly 70% coloration based on visual inspection were harvested weekly. In addition, at the end of the experiment, shoot fresh weight and stem diameter were measured in 29 plants of each treatment. The longest diameter of each plant was considered as its stem diameter. Water content and root-zone temperature were measured with frequency domain reflectometry (FDR)-based multi-sensors (GroSens, Grodan, The Netherlands). The FDR-based multi-sensor operates under the principles of dielectric constant measurement for water content monitoring and is integrated with the sensors for root-zone temperature and EC monitoring. Due to the limited number of FDR-based multi-sensor modules available in the experiment, the third incision level (L3) was excluded from the sensor assignment and three FDR-based multi-sensors were placed in the first, second, and fourth incision levels (L1, L2, and L4). The inside air temperature and relative humidity was measured using a climate sensor module in the center of the greenhouse (JN-DL1, JiNong, Republic of Korea). Water samples were extracted from the stone wool slabs using a 50 mL syringe to measure the dissolved oxygen variations following the protocol given in Bonachela et al. (2010). The collection points were randomly selected to ensure representative samples of the overall dissolved oxygen in the stone wool slabs. Five 10 mL samples of water were collected for each extraction (50 mL final volume). During 215–225 DAT, three measurements were recorded, and the extraction time was between 14:00 and 17:00.

### 2.4. Statistical Analysis

A completely randomized design was used, and each treatment was replicated five times and randomly distributed within the four gutter lines. The stone wool slabs at the beginning and end of the hanging gutter lines were excluded from the treatment application. Thus, five stone wool slabs and 30 plants were allocated for each treatment. One-way analysis of variance (ANOVA) was used to detect the difference in the cover incision effects on the dissolved oxygen, total tomato yield per plant, average fruit weight per plant, number of fruits per plant, shoot fresh weight, and stem diameter. We did not use two-way (time × treatments) repeated measure ANOVA since all incision treatments were simultaneously applied since the day of transplant. Thus, we analyzed all the measured data for the significance tests as independent samples. In addition to the significance tests, we performed normalization on the cumulative yield data to relatively compare and visualize the time course variation in the yield data between different treatments using the following equation:


(1) $$\begin{array}{l} x_{nor,t}=\frac{x_{t}-x_{min,t}}{x_{max,t}-x_{min,t}} \end{array}$$

where at any given harvesting time (*t*), *x*_*nor,t*_ is the normalized cumulative yield, *x*_*t*_ is the cumulative tomato yield to be normalized, *x*_*min,t*_ is the lowest cumulative yield among all the treatments, and *x*_*max,t*_ is the highest cumulative yield among all the treatments. The time-series analysis was conducted by visualizing the normalized yield change curves over the cultivation period. In addition, we investigated the yield response by treatments to air temperature variations by classifying the normalized yield distributions into temperature groups observed during the tomato harvest period. In addition, we investigated the yield response of each treatment to air temperature variations by classifying the normalized yield distributions into different temperature groups observed during the tomato harvest period.

## 3. Results

### 3.1. Root Environment

**Figure 2:**
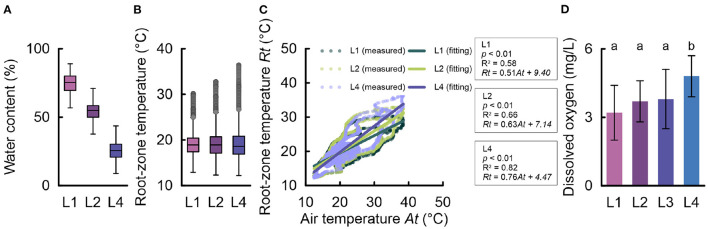
Changes in environmental root-zone factors: **(A)** water content distribution, **(B)** root-zone temperature distribution, **(C)** correlation between the air temperature and root-zone temperature, and **(D)** dissolved oxygen in the water extracted from the stone wool substrates during 215–255 days after transplanting (*n* = 15). Significance was analyzed using one-way ANOVA. The letters a and b denote significant differences between the cover incision treatments.

The environmental root zone properties were affected by the stone wool cover incision level (Figure 2). The L1 treatment was associated with the highest water content distribution in the stone wool slab (Figure 2A). The water content distribution associated with the L1 treatment differed greatly from that of the L4 treatment, which had the 100 cm cover incision and favorable drainage performance. The median water content of the L4 treatment was 26% (first quartile = 21%; third quartile = 30%), whereas that of the L1 treatment was 75% (first quartile = 70%; third quartile = 80%). With a median of 55% (first quartile = 50%; third quartile = 58%), the L2 water content value was between those of the L1 and L4 treatments.

In contrast to the water content variations, the root-zone temperature did not differ substantially, based on the median values (L1, 18.9°C; L2, 18.9°C; L4, 18.6°C) and quartile values (L1, first quartile = 17.4°C, third quartile = 20.4°C; L2, first quartile = 17.1°C, third quartile = 20.1°C; L4, first quartile = 16.9°C, third quartile = 20.8°C). However, distinct trends were observed for outlier values. The maximum outlier value of L1 was 30.2°C, while that of L4 was 36.5°C. Furthermore, a comparison of the root-zone temperatures of L1, L2, and L4 with concurrent variations in the greenhouse air temperature revealed that the slope of the correlation increased in the order L1 < L2 < L4 (L1, 0.50; L2, 0.63; L4, 0.76), along with the increase in the determination coefficients (L1, R^2^ = 0.58; L2, R^2^ = 0.66; L4, R^2^ = 0.82).

The dissolved oxygen level in the root-zone water of the L4 treatment was significantly higher than those of the other treatments. In L1, the dissolved oxygen average value was 34% below that of the L4 treatment (Figure 2D). The root-zone water of the L1 treatment had the lowest dissolved oxygen average value. However, the differences among the average values of L1, L2, and L3 were not significant. Although there was no significant difference, the dissolved oxygen average values of L2 and L3 were 14 and 17% higher than that of L1, respectively.

### 3.2. Biomass and Yield Components

**Figure 3:**
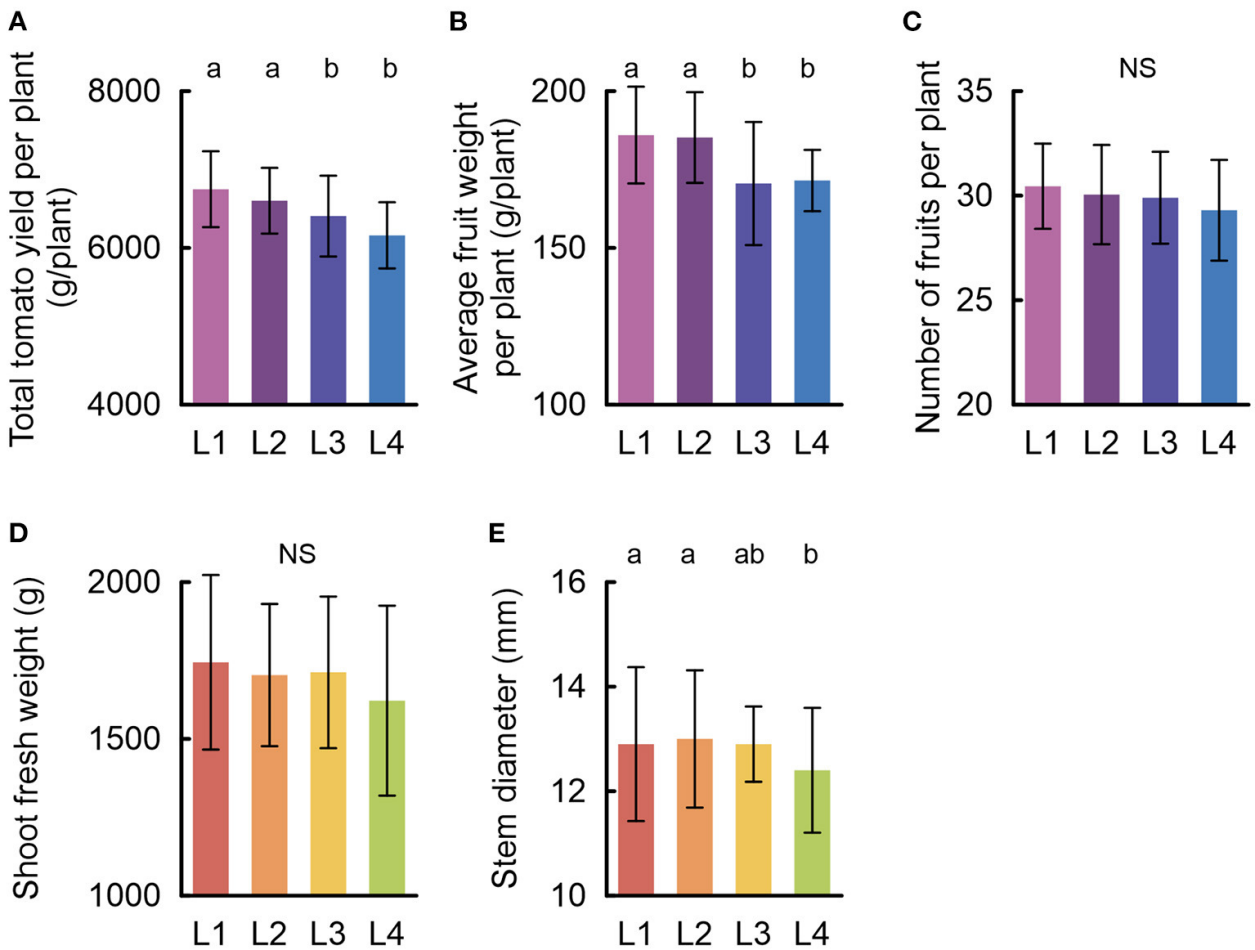
Growth performance of tomato cultivation (mean ± SD): **(A)** total tomato yield per plant (*n* = 20), **(B)** fruit weight (*n* = 20), **(C)** number of fruits per plant (*n* = 20), **(D)** shoot fresh weight (*n* = 29), and **(E)** stem diameter (*n* = 29). Significance was analyzed using one-way ANOVA. The letters a and b denote significant differences between the cover incision treatments. NS, no significant difference.

Significant growth performance differences were mainly grouped as L1–L2 and L3–L4 incision treatments (Figures 3A,B,E). The L1 treatment with the unfavorable drainage levels had the highest average yield, whereas the L4 treatment with the 100 cm cover incision and favorable drainage levels had the lowest average yield. The L1 treatment had a significantly higher average of 10% (*P* <0.05) tomato yield per plant than the L4 treatment (Figure 3A). Among the growth indices contributing to the tomato yield per plant, the number of fruits per plant and fresh weight of the shoots did not significantly vary between the treatments (Figures 3C,D), whereas significant differences were observed in the fruit weight and stem diameter (Figures 3B,E). Specifically, manipulation of the stone wool cover incision level increased the average fruit weight and average stem diameter by up to 8 and 4%, respectively. Apart from the statistically significant differences, an overall decrease in the averages of the growth indices was inversely correlated with an increase in the drainage performance level of the treatments (L1: limited cover incision lengths and drainage slit at a higher distance from the base of the slab; L2: limited cover incision lengths and drainage slit at a lowest part of the slab; L3: full opening on one side of the slab cover toward the gutter slope direction; L4: full opening in the lowest two longitudinal sides of the slab) (Figures 3A–E).

### 3.3. Trends in Normalized Yields Over Experimental Periods

**Figure 4:**
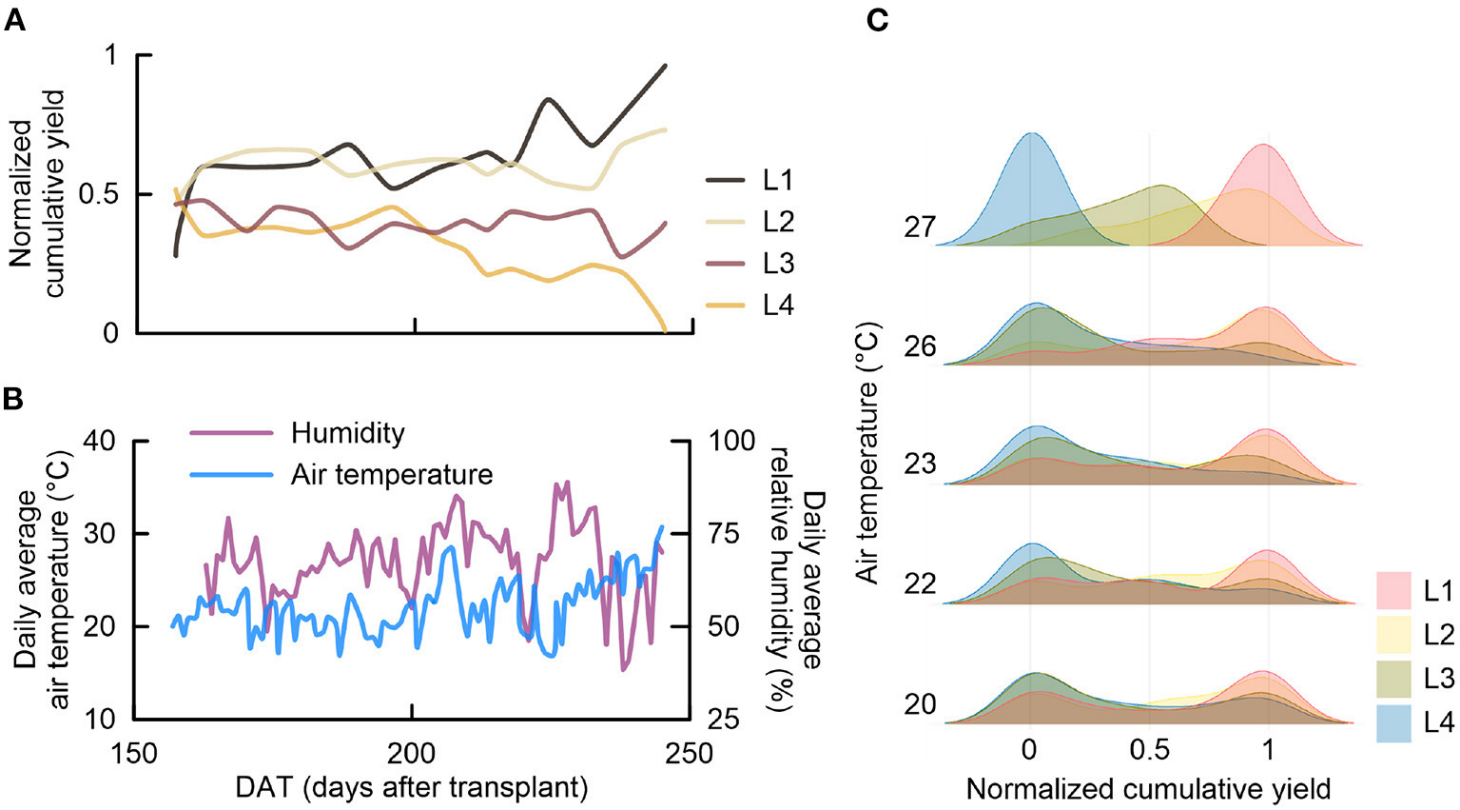
Trend in the normalized cumulative tomato yield and temperature variations during the experimental period: **(A)** average normalized cumulative yields (*n* = 20 per treatment) of the stone wool incision treatments, **(B)** average daily air temperature and relative humidity inside the greenhouse, and **(C)** normalized cumulative yield distribution (*n* = 20 per treatment) according to the air temperature groups observed during the tomato harvest period. L1, L2, L3, and L4, incision treatment levels; DAT, days after transplanting.

Normalization of tomato yield variations during the experimental period identified a time-dependency of relative changes associated with the stone wool cover incision treatments (Figure 4A). The trends in the normalized average yield during the second half of the experimental period were different from those during the first half of the observation period. At approximately 160 DAT, the normalized yields diverged between the treatments into two groups: L1 and L2 (>0.5) and L3 and L4 (<0.5). After the first divergence, the normalized yield fluctuated between the two groups. However, during the second half of the experimental period, the L1 treatment was associated with the largest tomato production increase until the end of the experiment. In contrast, the relative tomato production in the L4 treatment was reduced to the lowest level after the second half of the experimental period. The normalized yields at the end of the experiment were ranked in ascending order, as follows: L4 < L3 < L2 < L1.

A similar trend was observed for the daily average temperature inside the greenhouse (Figure 4B). Between 150 and 200 DAT, the daily average temperature and the relative humidity were maintained within 17–24°C and 38–89%, respectively. Beyond 200 DAT, the internal greenhouse temperature gradually increased to 31°C (Figure 4B). Based on the concurrent trends, the normalized yields of the different treatment samples (*n* = 20 per treatment) were assessed in combination with five average temperature ranges based on measurements during the harvest interval (Figure 4C). The ridgeline plot in Figure 4C illustrates the separation of the normalized tomato yield distribution according to the temperature ranges during the tomato harvest intervals. Within the temperature range of 20–26°C, the normalized yield values of each treatment sample were uniformly distributed. However, at 27°C, there was a clear separation between L1 and L4. Moreover, the L3-associated distribution moved toward the middle level, and the L2-associated distribution moved toward the upper-middle level.

## 4. Discussion

Stone wool cultivation is amenable to precision control. Various commercially available sensors can be utilized to maximize the efficacy of agronomic technologies (Neupane and Guo, 2019). However, there is a risk of compromising the performance of crop production and water use if the impact of system parameters is neglected. The plastic cover is an important component of the stone wool substrate system, affecting the air and water exchange at the atmosphere and slab interface by modulating its level of openness. However, the systemic effects associated with the plastic cover incision that could potentially interfere with the water use and crop production performance of stone wool-based agronomic techniques have not been studied. Our study is the first to report a series of environmental variations in the slab, aroused by the cover incision modulation and its subsequent impact on crop productivity. The results of this study will improve our understanding of the intertwined relationship between the stone wool cover incision, the environmental root-zone variables, and the associated yield variations.

Previously, the stone wool cover incisions were made based on the grower's expertise or using the methods recommended by the manufacturer, which was sufficient to achieve proper drainage function (Grodan, 2022). To date, the method of making an incision into the stone wool plastic cover for drainage has not been considered as a significant factor, as long as the drainage function is as expected. We found that the stone wool cover incision level modified the stone wool substrate parameters, which substantially affected environmental root-zone variables related to the hydraulic, thermal, and gaseous properties and significantly impacted crop production (Figures 2–4). Our results demonstrate that the stone wool system with high drainage performance could adversely affect its environmental rhizosphere characteristics.

The variations in water content and dissolved oxygen, in our study, demonstrate a general relationship between the liquid and gaseous phases in a porous substrate; increasing the water content in a porous substrate reduces the air content in the medium (Unger et al., 2009). Therefore, high water content can limit oxygen diffusion from the air-filled pores within the substrate (Yanful et al., 2006). Therefore, hours of long-term exposure of the plant roots to flooding conditions can eventually deplete the dissolved oxygen in the liquid phase (Ruperti et al., 2019). The water distribution in Figure 2A shows a clear distinction between the cover incision levels and the maximum achievable water content. Based on the general interactions, the maximum water capacity in this study is related to the field capacity, which is a core parameter of the hydraulic properties of the growth substrate (Assouline and Or, 2014). The plastic stone wool cover could not actually change the physicochemical properties of the slabs; thus, we do not suggest that the cover incision modifies the hydraulic properties of the stone wool slab itself. However, the alterations of the achievable maximum water content triggered by different cover incision levels modified the thermal properties of the stone wool slabs. As shown in Figure 2B, the cover incision treatment affected the outlier temperatures of the root-zone temperature distribution. Although the median and quartile values were similar, the maximum outlier values were 30.2°C in L1 and 36.5°C in L4, exceeding the optimal root zone temperature (<25°C) (Tindall et al., 1990). Typically, the heat inside a greenhouse is induced by solar radiation and subsequently transferred to root-zone (Fitz-Rodríguez et al., 2010). Water, air, and stone wool materials are coexisting components of the root zone that differ in their heat capacity, with water having the highest heat capacity (Rubio et al., 2015). In addition, the root biomass itself is a significant component within the slab, with its own thermal properties. Therefore, the greater water content increased the thermal mass of the slab, conferring a root zone buffer to changes in air temperature.

Specifically, the tendency for increased outlier values could be derived from the elevated susceptibility to the thermal peak during the cultivation period due to a lower heat capacity and water distribution range. This is supported by our data; the correlation test between the greenhouse air temperature and the stone wool temperatures for each treatment identified different sensitivities to the thermal variations of the greenhouse air temperature (Figure 2C). L4, with the favorable drainage performance, had a high positive correlation and slope with air temperature. The slope and coefficient of determination decreased in descending order from L4 to L1. The high correlation associated with L4 suggested rapid responsiveness of the stone wool substrate under this treatment. Consequently, the incision level modifications of the stone wool substrate cover altered the hydraulic, thermal, and gaseous properties of the plastic wrapped stone wool slabs, resulting in significant changes in tomato production (Figure 3). The L1 treatment was applied as an incision level with poor drainage performance. Although water-logged condition could be an unfavorable for normal growth (Striker, 2012), high water conditions, in our study, did not result in hypoxia symptoms such as growth retardation (Fukao and Bailey-Serres, 2004) or a significant yield reduction. On the contrary, the L1 treatment was associated with the highest yield. Although the dissolved oxygen in the water extracted from the stone wool substrate indicated the lowest average value for L1, there were no significant differences between this treatment and the L2 and L3 treatments, which had normal drainage conditions (Grodan, 2022) (Figure 2D). Hypoxia development is a function of exposure time and oxygen conditions (Ruperti et al., 2019). Irrigation is frequently supplied in soilless culture systems (Ta et al., 2012; Shin and Son, 2016). Therefore, oxygen consumption in the root zone might be replenished by a series of irrigation events. Under these experimental conditions, high water content did not act as an inhibitory condition; on the contrary, it significantly enhanced plant growth parameters, such as stem diameter and fruit weight, increasing the tomato production linked to L1 treatment. These results are also supported by previous studies demonstrating the effects of root-zone water content on the plant stem diameter (Nortes et al., 2005; Li et al., 2012).

Root-zone temperature is an important yield-impacting environmental component, other than the water content and dissolved oxygen. In general, the root-zone temperature and yield response have a quadratic curve relation (Díaz-Pérez et al., 2005). Based on this relationship, plant growth can be increased up to the vertex of the curve and decreased thereafter. The normalized yield trends associated with temperature ranges during the experimental period suggested that differences in the heat capacity affected the tomato growth (Figure 4). This finding also suggests a trade-off between drainage performance and heat capacity in stone wool substrate systems during high-temperature weather. However, in this study, we have used a single stone wool product. Therefore, although stone wool products have similar physicochemical characteristics, the variations in the different stone wool products should be additionally considered.

## 5. Conclusions

The cover incision method significantly affected the average water content of the stone wool substrate, which is a predictable response typically expected by the modified drainage performance. However, we noted that this process could entail variations in the thermal sensitivity of the stone wool substrate with yield responses to water management. This result reveals another trade-off factor when considering the modulation of drainage and gas exchange performance in a stone wool-based system. Although the difference in average total tomato yield per plant between the highest (L4; 6,160 g/plant) and lowest drainage performance (L1; 6,749 g/plant) treatment was ~589 g/plant, significant differences at the individual plant level can be amplified into very large differences on a commercial scale. Our study reported a series of environmental changes and the resulting crop growth impacts that were not intuitively expected even for a relatively standardized and simplified system such as stone wool slab. In particular, we showed that generally unfavorable drainage conditions can be exploited as a beneficial buffer to changes in air temperature during hot periods. The expanded understanding of the effect associated with the cover incision provides insight into further optimization of water use and crop production in stone wool-based water management systems. For example, monitoring dissolved oxygen concentration, root zone temperature, and air temperature and modeling the relationship between these variables and drainage performance can be helpful for designing a more dynamic stone wool water management program that reduces the cooling energy load during the high-temperature periods. Further, for the technical aspects, developing a drainage performance adjustable gutter might be more effective for implementing this management model.

## Data Availability Statement

The raw data supporting the conclusions of this article will be made available by the authors, without undue reservation.

## Author Contributions

TA and JL conceived and supervised the study. J-SY designed and performed the laboratory analysis. Y-HI and YY analyzed the data. TA wrote the manuscript. All authors approved the final version of the manuscript.

## Funding

This work was supported by National R&D Program through the National Research Foundation funded by Ministry of Science and ICT (2020M3A9I3037807 and Project No. 1711156197), the Korea Institute of Planning and Evaluation for Technology in Food, Agriculture and Forestry (IPET) and the Korea Smart Farm R&D Foundation (KosFarm) through the Smart Farm Innovation Technology Development Program, funded by the Ministry of Agriculture, Food and Rural Affairs (MAFRA) and Ministry of Science and ICT (MSIT), and the Rural Development Administration (RDA) (Nos. 421006-03-2-HD040-KIST and 421039-03-2-HD020-KIST).

## Conflict of Interest

The authors declare that the research was conducted in the absence of any commercial or financial relationships that could be construed as a potential conflict of interest.

## Publisher's Note

All claims expressed in this article are solely those of the authors and do not necessarily represent those of their affiliated organizations, or those of the publisher, the editors and the reviewers. Any product that may be evaluated in this article, or claim that may be made by its manufacturer, is not guaranteed or endorsed by the publisher.

## References

[B1] Ahn T. I. Yang J.-S. Park S. H. Moon H. W. Lee J. Y. (2020). Translation of irrigation, drainage, and electrical conductivity data in a soilless culture system into plant growth information for the development of an online indicator related to plant nutritional aspects. Agronomy 10:1306. 10.3390/agronomy10091306

[B2] Assouline S. Or D. (2014). The concept of field capacity revisited: defining intrinsic static and dynamic criteria for soil internal drainage dynamics. Water Resour. Res. 50, 4787–4802. 10.1002/2014WR015475

[B3] Bar-yosef B.. (2008). “Chapter 9: Fertigation management and crops response to solution recycling in semi-closed greenhouses,” in Soilless Culture, eds M. Raviv and J. H. Lieth (Amsterdam: Elsevier), 341–424. 10.1016/B978-044452975-6.50011-3

[B4] Bonachela S. Quesada J. Acuña R. A. Magán J. J. Marfà O. (2010). Oxyfertigation of a greenhouse tomato crop grown on rockwool slabs and irrigated with treated wastewater: oxygen content dynamics and crop response. Agric. Water Manage. 97, 433–438. 10.1016/j.agwat.2009.10.016

[B5] Bougoul S. Boulard T. (2006). Water dynamics in two rockwool slab growing substrates of contrasting densities. Sci. Hortic. 107, 399–404. 10.1016/j.scienta.2005.11.007

[B6] Bougoul S. Ruy S. de Groot F. Boulard T. (2005). Hydraulic and physical properties of stonewool substrates in horticulture. Sci. Hortic. 104, 391–405. 10.1016/j.scienta.2005.01.018

[B7] Bussell W. T. Mckennie S. (2004). Rockwool in horticulture, and its importance and sustainable use in New Zealand. N. Z. J. Crop Hortic. Sci. 32, 29–37. 10.1080/01140671.2004.9514277

[B8] de Koning A. Tsafaras I. (2017). Real-time comparison of measured and simulated crop transpiration in greenhouse process control. Acta Hortic. 1170, 301–308. 10.17660/ActaHortic.2017.1170.36

[B9] De Rijck G. Schrevens E. (1998). Distribution of nutrients and water in rockwool slabs. Sci. Hortic. 72, 277–285. 10.1016/S0304-4238(97)00144-1

[B10] Díaz-Pérez J. C. Phatak S. C. Giddings D. Bertrand D. Mills H. A. (2005). Root zone temperature, plant growth, and fruit yield of tomatillo as affected by plastic film mulch. HortScience 40, 1312–1319. 10.21273/HORTSCI.40.5.1312

[B11] Fitz-Rodríguez E. Kubota C. Giacomelli G. A. Tignor M. E. Wilson S. B. McMahon M. (2010). Dynamic modeling and simulation of greenhouse environments under several scenarios: a web-based application. Comput. Electron. Agric. 70, 105–116. 10.1016/j.compag.2009.09.010

[B12] Fukao T. Bailey-Serres J. (2004). Plant responses to hypoxia?is survival a balancing act? Trends Plant Sci. 9, 449–456. 10.1016/j.tplants.2004.07.005 15337495

[B13] Grodan (2022). Handling and Placing of the Slabs. Available online at: https://www.grodan.com/siteassets/downloads/tools-services/english/ts-3-3-handling-the-slabs-en.pdf?f=20190104043133.

[B14] Gruda N. S.. (2019). Increasing sustainability of growing media constituents and stand-alone substrates in soilless culture systems. Agronomy 9:298. 10.3390/agronomy9060298

[B15] Jensen M. H.. (1997). Hydroponics. HortScience 32, 1018–1021. 10.21273/HORTSCI.32.6.1018

[B16] Lee A.. (2010). Water and EC management. Pract. Hydrop. Greenhouses 111, 48–54. Available online at: https://search.informit.org/doi/10.3316/informit.772380591933400

[B17] Li Y.-J. Yuan B.-Z. Bie Z.-L. Kang Y. (2012). Effect of drip irrigation criteria on yield and quality of muskmelon grown in greenhouse conditions. Agric. Water Manage. 109, 30–35. 10.1016/j.agwat.2012.02.003

[B18] Montesano F. F. van Iersel M. W. Boari F. Cantore V. D'Amato G. Parente A. (2018). Sensor-based irrigation management of soilless basil using a new smart irrigation system: effects of set-point on plant physiological responses and crop performance. Agric. Water Manage. 203, 20–29. 10.1016/j.agwat.2018.02.019

[B19] Neupane J. Guo W. (2019). Agronomic basis and strategies for precision water management: a review. Agronomy 9:87. 10.3390/agronomy9020087

[B20] Nortes P. Pérez-Pastor A. Egea G. Conejero W. Domingo R. (2005). Comparison of changes in stem diameter and water potential values for detecting water stress in young almond trees. Agric. Water Manage. 77, 296–307. 10.1016/j.agwat.2004.09.034

[B21] Rubio E. Agustina B. Marín M. Bericua A. (2015). Cooling systems based on cold compressed air: a review of the applications in machining processes. Manuf. Eng. Soc. Int. Conf. 132, 413–418. 10.1016/j.proeng.2015.12.513

[B22] Ruperti B. Botton A. Populin F. Eccher G. Brilli M. Quaggiotti S.. (2019). Flooding responses on grapevine: a physiological, transcriptional, and metabolic perspective. Front. Plant Sci. 10:339. 10.3389/fpls.2019.00339 30972087 PMC6443911

[B23] Saha U. K. Papadopoulos A. P. Hao X. Khosla S. (2008). Irrigation strategies for greenhouse tomato production on rockwool. HortScience 43, 484–493. 10.21273/HORTSCI.43.2.484

[B24] Saiful Islam F. Hirai H. Kitaya Y. (2008). Hydroponic cultivation of carrots using modified rockwool blocks. J. Appl. Hortic. 10, 132–136. 10.37855/jah.2008.v10i02.28

[B25] Shin J. H. Son J. E. (2016). Application of a modified irrigation method using compensated radiation integral, substrate moisture content, and electrical conductivity for soilless cultures of paprika. Sci. Hortic. 198, 170–175. 10.1016/j.scienta.2015.11.015

[B26] Sonneveld C.. (1991). “Rockwool as a substrate for greenhouse Crops,” in High-Tech and Micropropagation I, ed Y. P. S. Bajaj (Berlin; Heidelberg: Springer Berlin Heidelberg), 285–312. 10.1007/978-3-642-76415-8_17

[B27] Striker G.. (2012). “Chapter 1: Flooding stress on plants: anatomical, morphological and physiological responses,” in Botany, ed J. K. Mworia (Rijeka: IntechOpen), 3–28.

[B28] Ta T. H. Shin J. H. Noh E. H. Son J. E. (2012). Transpiration, growth, and water use efficiency of paprika plants (*Capsicum annuum* L.) as affected by irrigation frequency. Hortic. Environ. Biotechnol. 53, 129–134. 10.1007/s13580-012-0095-2

[B29] Tindall J. A. Mills H. Radcliffe D. (1990). The effect of root zone temperature on nutrient uptake of tomato. J. Plant Nutr. 13, 939–956. 10.1080/01904169009364127

[B30] Unger I. M. Motavalli P. P. Muzika R.-M. (2009). Changes in soil chemical properties with flooding: a field laboratory approach. Temp. Agrofor. 131, 105–110. 10.1016/j.agee.2008.09.013

[B31] Van Noordwijk M.. (1990). “Synchronisation of supply and demand is necessary to increase efficiency of nutrient use in soilless horticulture,” in Plant Nutrition?Physiology and Applications: Proceedings of the Eleventh International Plant Nutrition Colloquium, ed M. L. van Beusichem (Wageningen; Dordrecht: Springer Netherlands), 525–531. 10.1007/978-94-009-0585-6_87

[B32] Yanful E. K. Morteza Mousavi S. De Souza L.-P. (2006). A numerical study of soil cover performance. J. Environ. Manage. 81, 72–92. 10.1016/j.jenvman.2005.10.006 16556481

[B33] Zhao Y. Zhang Q. Li J. Yan X. He H. Gao X. Jia G. (2021). High temperature in the root zone repressed flowering in Lilium × formolongi by disturbing the photoperiodic pathway and reconfiguring hormones and primary metabolism. Environ. Exp. Bot. 192:104644. 10.1016/j.envexpbot.2021.104644

